# Children can discriminate the authenticity of happy but not sad or fearful facial expressions, and use an immature intensity-only strategy

**DOI:** 10.3389/fpsyg.2015.00462

**Published:** 2015-05-05

**Authors:** Amy Dawel, Romina Palermo, Richard O’Kearney, Elinor McKone

**Affiliations:** ^1^Research School of Psychology and ARC Centre of Excellence in Cognition and its Disorders, The Australian National University, Canberra, ACT, Australia; ^2^Research School of Psychology, The Australian National University, Canberra, ACT, Australia; ^3^ARC Centre of Excellence in Cognition and its Disorders, and School of Psychology, University of Western Australia, Perth, WA, Australia

**Keywords:** facial emotion, genuine, posed, Duchenne, empathy

## Abstract

Much is known about development of the ability to label facial expressions of emotion (e.g., as happy or sad), but rather less is known about the emergence of more complex emotional face processing skills. The present study investigates one such advanced skill: the ability to tell if someone is genuinely feeling an emotion or just pretending (i.e., authenticity discrimination). Previous studies have shown that children can discriminate authenticity of happy faces, using expression intensity as an important cue, but have not tested the negative emotions of sadness or fear. Here, children aged 8–12 years (*n* = 85) and adults (*n* = 57) viewed pairs of faces in which one face showed a genuinely-felt emotional expression (happy, sad, or scared) and the other face showed a pretend version. For happy faces, children discriminated authenticity above chance, although they performed more poorly than adults. For sad faces, for which our pretend and genuine images were equal in intensity, adults could discriminate authenticity, but children could not. Neither age group could discriminate authenticity of the fear faces. Results also showed that children judged authenticity based on intensity information alone for all three expressions tested, while adults used a combination of intensity and other factor/s. In addition, novel results show that individual differences in empathy (both cognitive and affective) correlated with authenticity discrimination for happy faces in adults, but not children. Overall, our results indicate late maturity of skills needed to accurately determine the authenticity of emotions from facial information alone, and raise questions about how this might affect social interactions in late childhood and the teenage years.

## Introduction

Developmental studies of facial expression processing have focused almost exclusively on children’s ability to label emotional facial expressions (i.e., as happy vs. sad etc.; for review see Widen, 2013). Yet being able to name the facial expression being displayed is not enough for successful social interaction. It is also important to be able to tell whether a facial display matches a person’s underlying emotional experience (i.e., a genuine expression) or not (i.e., a posed expression). Concerning this ability, previous developmental studies have focused on happy facial expressions. None have investigated any negative facial expressions using stimuli for which it has been confirmed that adults can discriminate authenticity. Here we test for the first time children’s ability to discriminate the authenticity of two negative facial expressions: sadness and fear, as well as happiness. We also provide the first test of the association between children’s authenticity discrimination and perceived intensity of the expression across all three emotions. Finally, we provide the first evidence of correlations between individual differences in empathy and typical adults’ authenticity discrimination ability, and test the same correlation in children.

### Genuine and Posed Expressions

Being able to tell the difference between genuine and posed facial expressions is crucial to social interaction because the two types of expression carry different meanings and imply different social responses. For example, if a person sees someone they know from school or work in a busy mall, a genuine smile might signal an invitation to approach and chat, whereas a posed only-being-polite smile might signal that further social interaction is not wanted at this time. In another example concerning sad expressions, not being able to tell the difference between genuine and posed sadness might increase vulnerability to manipulation: somebody showing a pretend sad expression could use it to elicit help from somebody who cannot tell the sadness is faked.

Genuine and posed expressions differ in several ways. The fundamental and critical distinction is that genuine expressions correspond with a congruent underlying emotion (e.g., smiling when feeling happy, frowning when feeling angry), whereas posed expressions do not. Here we investigate specifically the type of posed expressions that are *pretend*, in which there is no strong underlying experience of any emotion, such as smiling for a photograph, or playing pretend with a child whilst feeling emotionally neutral. (Note these potentially differ from posed expressions that are *masked*, in which the underlying emotion is *incongruent* with the facial display, e.g., masking anger using a smile; Gosselin et al., 2002a).

As a consequence of the differences in underlying emotional experience, genuine and posed facial expressions may also differ in their physical appearance, providing perceivers with some clues about emotional authenticity. Although the nature of these physical differences is not yet fully understood, some differences have been identified. One approach has been to use the Facial Action Coding System (FACS; Ekman et al., 2002), which is a tool for objectively measuring the degree of activation of different facial muscle groups, termed action units (AUs). Genuine expressions sometimes include so-called “reliable” AUs (Ekman, 2003), which occur less often in posed expressions (Ekman et al., 1988), and which people have less ability to control voluntarily (Mehu et al., 2012; although note that some people are able to voluntarily activate these AUs, Gunnery et al., 2012). The best established of these is AU6 for happy, or the “Duchenne” marker, which involves contraction of the orbicularis oculi muscle around the eyes to form wrinkles. AU6 has been associated with genuine happy expressions (e.g., Ekman et al., 1988; at least for Caucasians, Thibault et al., 2012). Reliable AUs for other emotions are less well established, but for sadness the AU1+4 combination (proposed by Ekman, 2003), which pulls the medial portion of the brow upward and together, has recently been empirically associated with genuine sadness (McLellan et al., 2010; although see Mehu et al., 2012, who found *observers* associated AU23 with authentic sadness, and not AU1+4). Note that for fear, it has not been empirically established in the literature what, if any, are the reliable AUs for genuine fear (Ekman, 2003). In addition to reliable AUs, other physical differences may include symmetry and signs of arousal. Genuine expressions are thought to be more symmetrical than posed expressions (Frank and Ekman, 1993; Ekman, 2003), and may include physical signs of arousal such as pupil dilation or skin “blushing” (Levenson, 2014), which are missing from pretend expressions because there is minimal underlying emotional arousal. Finally, intensity (how weak or strong the expression is) may potentially differ between genuine and posed expressions, particularly for happy where it has been suggested that a stronger underlying experience of happiness results in a more intense facial display (Hess et al., 1995).

Stimuli used in facial authenticity studies have been generated in several different ways. For happy in particular, some researchers have defined genuine happy expressions as any smile that includes AU6, and pretend happy expressions as any smile that does not include AU6 (e.g., Beaupré and Hess, 2003). Typically, these stimuli have been generated using actors who are able to voluntarily activate AU6. However, whether these actors were feeling underlying happiness is unknown and thus, although these stimuli mimic the muscle AU characteristics of genuine and pretend happiness, they may not include other physical markers of authenticity, such as signs of arousal. Given this, we suggest it is also valuable to test stimuli in which the emotional state of the photographed person is known to correspond to the assigned status of the stimulus as genuine versus posed.

**FIGURE 1:**
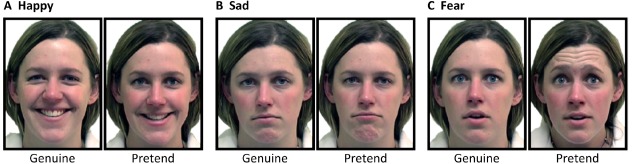
**Examples of genuine and pretend (A) happy, (B) sad and (C) fear expressions (McLellan et al., 2010)**.

For this reason, in the present study, we use genuine expression stimuli from McLellan and colleagues (e.g., McLellan et al., 2010; see Figure 1 for examples). These stimuli were elicited in a laboratory setting using procedures developed by Miles (2005). For genuine expressions, emotions were elicited by looking at emotional pictures, listening to emotional sounds (e.g., baby laughing), or remembering an emotional event, and subsequently were verified by self-report of the people who had displayed the expressions to correspond with their underlying experience of emotion. The McLellan stimuli also include pretend versions of the same expressions, from the same models, which were elicited by instructing stimulus models to pose or pretend a sad or fearful face, fake a fearful reaction, or smile for a license photo, and which were subsequently verified by self-report to have been generated without any strong underlying experience of emotion. For the happy and sad expressions, the genuine versions include AU6 (happy) or AU1+4 (sad), while these reliable-AU markers are absent in the pretend versions. (For fear, reliable-AU status of the stimuli cannot be determined given that reliable-AUs for fear have not been established).

### Adult Authenticity Discrimination Ability for the McLellan Stimuli

For the present study, we wished to select stimuli, and emotions, for which previous studies have found adult authenticity discrimination performance was above chance (thus allowing the examination of developmental trends). For adults, five published studies using stimuli from McLellan have tested whether observers can distinguish genuine from pretend expressions (McLellan et al., 2010, 2012; Johnston et al., 2011; Douglas et al., 2012; McLellan and McKinley, 2013; note that all of these studies did not use exactly the same stimulus items). Participants were instructed, “Your job is to decide… whether or not they are [the person shown is] feeling each emotion. For instance, sometimes when people smile it does not necessarily mean that they are actually feeling happy.” (McLellan et al., 2010, p. 1283). Participants were then asked to give a yes/no response to the question “Are the following people *feeling* [emotion]?” For happiness and sadness, all five studies (McLellan et al., 2010, 2012; Johnston et al., 2011; Douglas et al., 2012; McLellan and McKinley, 2013) found that adults were significantly above chance at authenticity discrimination on this task (i.e., more “yes” responses for genuine expressions than for pretend expressions as indicated by A’, a non-parametric signal detection score that combines hits and false alarms). For fear, of the two studies that tested this emotion, the initial study found significant if weak discrimination (McLellan et al., 2010), although this was not replicated in a later study (which found no discrimination; Douglas et al., 2012). For other expressions, there have either been no tests of McLellan-type stimuli (anger, surprise), or no evidence of above-chance authenticity discrimination in adults (e.g., disgust; Douglas et al., 2012). Overall then, there is good evidence adults can discriminate the authenticity of the McLellan happy and sad expression stimuli, with equivocal evidence regarding fear expressions.

### Previous Studies of Authenticity Discrimination in Development

Turning to development, there is evidence even very young children have in place at least some of the abilities needed to determine the genuineness of others’ emotional signals. Testing multimodal signals of emotions—specifically, adults communicating an emotion simultaneously through facial expression, body gestures, and voice—Walle and Campos (2014) showed that 19-month-olds can detect incongruency between the emotional display and the context of the rest of an event (e.g., a parent displaying pain, while hitting a hammer not on their finger but on the table nearby; although note that certain aspects of emotion–context interactions are not mature even by 12 years of age, Dawel et al., 2015) and can detect incongruency between two successive emotions (e.g., an actress displaying disgust followed immediately by happiness). These infants also showed sensitivity to whether a multimodal expression of fear was of normal versus exaggerated intensity. Importantly, however, in the Walle and Campos (2014) study all scenarios were acted (i.e., all facial expressions were likely posed rather than genuinely-felt) and the study concerned ability to discern authenticity-related information from multimodal stimuli, not facial information alone, which is the focus of the present investigation.

Studies that have tested specifically the ability to determine the authenticity of facial expressions, by contrasting genuine and posed versions of the expression, have tested children rather than infants. We are aware of six such studies. Five tested happy expressions, but only one tested any other emotion.

For happy, four studies varied authenticity by creating stimuli using the AU6 present–absent method (Gosselin et al., 2002b, 2010; Del Giudice and Colle, 2007; Thibault et al., 2009). One used happy faces created using the Miles/McLellan method where the subjective feelings of the photographed person are known (Blampied et al., 2010). Results of both methods agree that children can discriminate happy authenticity above chance from as young as 4 years of age, but do not reach adult levels of performance even by 16–17 years of age. Two of these studies also investigated the physical cues that children use to achieve authenticity discrimination in happy faces. Thibault et al. (2009) used smile stimuli that varied in intensity of the smile, and were also either with, or without, the reliable-AU marker for genuine happy AU6. From their data, Thibault et al. (2009) concluded that children from 4 years of age used intensity of the expression to judge authenticity, and also the presence of AU6. Del Giudice and Colle (2007) examined the relationship between 8-year-olds’ judgments of smile authenticity and FACS-coded AU intensity. This study found that expressions were judged by 8-year-olds as more authentic if they included bare-teethed smiles (AU25), stronger activation of AU6, and/or stronger activation of the “lid tightener” (AU7), which is easily confusable with AU6. Their results suggest that it may not be not the presence of AU6 per se that children use to judge smile authenticity, but rather the increased intensity of the expression that is associated with activation of AU6 (or AU7).

For other emotions, the only previous study videoed children’s genuine reactions to “disliked” stimuli (most commonly producing expressions of disgust, e.g., tasting a salty drink; Soppe, 1988). Child observers (6–12 year olds) could not discriminate these above chance from pretend dislike reactions to neutral stimuli. Unfortunately, this finding is difficult to interpret as evidence regarding developmental trends because adults also could not discriminate authenticity of the same stimuli. Thus overall, there have been no studies that have tested children’s ability to discriminate emotion authenticity of negative facial expressions for which adults have successfully demonstrated authenticity discrimination.

### Present Study

The primary aim of the present study was to provide the first test of children’s ability to discriminate the authenticity of two negative facial expressions, sad and fear, as well as happy expressions, in 8–12 year olds relative to adults. We tested 8–12 year olds because, by 8 years of age, children have a good conceptual understanding of the difference between genuine and pretend expressions (Sidera et al., 2011). We tested sad and fearful expressions specifically because these were the only two negative expressions for which we were able to obtain genuine-pretend stimulus pairs from the same identity models (created using the Miles/McLellan method), and for which adults had already demonstrated ability to discriminate authenticity (for sad, consistently above chance in five studies), or at least some evidence of ability to discriminate authenticity (for fear, above chance in one out of the two previous studies). We also included happy faces because it is well established that children of the age tested here can discriminate their authenticity, which allowed us to use happy to validate our task (i.e., children’s above-chance performance for happy expressions would help us to establish that children understood the task). We used a task that presents pairs of faces, rather than individual faces (e.g., as in Blampied et al., 2010; McLellan et al., 2010), to minimize task demands for children (Gosselin et al., 2010). In our two-alternative-forced-choice (2-AFC) paradigm, participants were shown pairs of genuine and pretend expressions from the same model and asked to decide which of the pair was “only pretending.”

A second aim of the present study was to obtain information relevant to understanding the strategies children use to discriminate authenticity, and particularly the extent to which they rely only on intensity of the facial expression, or a combination of intensity and other factors. Previous studies of this question have examined only the expression of happy. Here, we examine relationships between authenticity discrimination and perceived expression intensity, across all three expressions of happy, sad, and fear, to determine the contribution of intensity to children’s judgments of authenticity more broadly, beyond just the expression of happy. In addition, the analysis we use (see Results) allows us to determine whether children, and adults, demonstrate significant use of cues *beyond* intensity. Note that our stimuli do not, in general, allow us to define the specific nature of such cues (e.g., to what extent they include reliable-AUs versus other physical differences that might be present between the genuine and posed McLellan faces). Certain outcome possibilities, however, would allow us to draw some limited conclusions regarding other cues (e.g., for sad, the genuine but not posed expressions contain AU1+4; thus, a finding that, say, adults can discriminate authenticity of these stimuli above chance while children cannot would imply that children do *not* use this reliable-AU).

Our final aim was to examine associations between authenticity discrimination ability and individual differences in empathy. This is a largely novel question even in adults, and has not previously been tested at all in children. That there might be such an association is suggested by some theoretical models of empathy that link perception and action, so that by perceiving another’s situation the observer creates some kind of simulation, either through emotional or motoric representation, of the other’s situation that results in sharing of their emotional experience [e.g., theories that empathy is derived from emotional contagion, see Maibom, 2012; or the perception-action model of empathy (PAM), Preston and de Waal, 2002]. These theories do not specifically discuss a relationship between empathy and ability to determine *authenticity* of facial emotion, but do make it plausible that such an association could exist. For example, we suggest an association with authenticity discrimination might arise from a simulation process either because people with greater simulation abilities might be *better* at discriminating between genuine and pretend expressions because they experience an especially strong emotional experience in response to genuine expressions (predicting a positive correlation), or, in the opposite direction, that they might be *worse* at authenticity discrimination because they experience an indiscriminately strong emotional experience to both genuine and pretend expressions (predicting a negative correlation; Manera et al., 2013). These predictions regarding simulation appear to relate more specifically to the *affective* component of empathy—the extent to which a person is emotionally responsive to others’ experiences (e.g., the extent to which they feel sad, sympathetic or distressed because a friend is crying). In the present study, we also examine *cognitive empathy*—the ability to infer what another person is thinking and feeling from physical cues in the face and body, contextual information, and knowledge of the person (e.g., using a frown to infer that someone is angry; see Maibom, 2012). As cognitive and affective empathy are at least partly independent facets (Jolliffe and Farrington, 2006; Dadds et al., 2008), we examined them separately. Concerning predictions for cognitive empathy, we suggest that, potentially, observers could use cognitive strategies (e.g., explicit knowledge of the AU6 marker or arousal cues) to infer authenticity, predicting a positive correlation between cognitive empathy and authenticity discrimination. Regarding previous empirical tests, we are aware of only one previous study that has examined associations between empathy and authenticity discrimination in adults (but cf. Manera et al., 2013, for study of associations between emotional contagion and authenticity discrimination). McLellan and McKinley (2013) found a positive correlation between empathy (affective and cognitive components combined) and authenticity discrimination within a clinical traumatic brain injury group; however, this correlation was not significant within neurologically healthy controls with a small sample size (*n* = 19). Here, we re-examine this association within typically developing adults and, for the first time, test it in children.

## Materials and Methods

### Participants

Participants analyzed were 85 children (*M*_age_ = 10.0 years, SD_age_ = 1.1, age range = 8.3–12.3, 46 females) and 57 young adults (*M*_age_ = 19.3, SD_age_ = 2.2, age range = 17–27, 40 females). All participants were Caucasian, to match the race of face stimuli, because there are race-related cultural differences in the perception of expression authenticity (e.g., some non-Caucasian cultures do not interpret AU6 as a sign of genuine happiness; Thibault et al., 2012). Adults were recruited via fliers posted around campus at the Australian National University. Children were from two local primary schools, and were recruited by having the schools send letters home to all parents in the class requesting their child’s participation in the study. All participants were reported to have normal or corrected-to-normal vision. Informed, written consent was obtained from adult participants and from the parents of child participants. Verbal assent was also obtained from child participants. Adults were paid $15 per hour for their participation, or given undergraduate course credit. Children were rewarded with certificates and stickers. This study was approved by, and conducted in accordance with the guidelines of, the Human Research Ethics Committee at The Australian National University. Additionally, for children, approval for the study was obtained from the ACT Government Education and Training Directorate.

We excluded data from seven additional participants who, on a screening questionnaire, reported major disorders that can affect face processing (e.g., brain injury, Autism, etc.). These were five children reported by parents to have an intellectual impairment (1), ADHD or ADD (3), or Aspergers disorder (1), and two adults who reported epilepsy (1) or severe migraines with aura (1).

### Session Structure and Order of Tasks

Participants were tested in a single session lasting up to one hour (children) or one and a half hours (adults, extra time was for completing questionnaires and, for the first *n* = 26 adults tested, the emotion labeling and intensity rating tasks). Tasks reported in the present article were completed as part of a larger battery, but always in the following order: basic emotion labeling task (i.e., categorizing the facial expression as happy, sad, etc.); authenticity discrimination task; intensity rating task (adults only; note that children’s ratings of affective stimuli tend correlate highly with adults’ ratings, *r*s > 0.82; McManis et al., 2001); and finally demographic, screening, and empathy questionnaires (for adults; for children the questionnaires were completed by parents prior to the session). Experimental tasks were run using Macintosh computers. Faces were presented on an attached ELO IntelliTouch touchscreen with screen size 15” and resolution 1024 × 768, using Superlab version 4.0 software. Participants responded by touching the screen.

### Facial Expression Stimuli

Examples of the facial expression stimuli are shown in Figure 1. Stimuli were genuine and pretend versions of happy, sad and fear expressions. In total, there were 12 genuine-pretend pairs; 4 happy, 4 sad, and 4 fearful (24 face images total). The stimuli were provided by McLellan (personal communication, 2011), comprising a set that largely overlapped with that used by McLellan et al. (2010), and all were created in the manner described in that article [i.e., following Miles (2005) as described in the final paragraph of “Genuine and Posed Expressions” in our Introduction]. Genuine and pretend versions of each emotion were displayed by four female stimulus models (all three expressions = 1 model; happy and sad = 2 models; happy and fear = 1 model; sad only = 1 model; fear only = 2 models). Stimulus models were from the general population, and did not have any specific training. Faces were displayed centrally, and subtended 5.5 × 7.3° visual angle (4.8 cm wide × 6.4 cm high at the viewing distance of approximately 50 cm).

### Emotion Labeling

The 24 happy, sad and fear face stimuli were presented individually in random order for each participant, intermixed with 16 additional images displaying genuine and pretend anger and disgust expressions to make the labeling task a 5-choice response (total stimuli = 40 faces). (Anger and disgust stimuli were also provided by McLellan and colleagues; note these were not included in our authenticity discrimination task). The task was to indicate what expression each face was displaying by choosing from the five emotion labels presented onscreen (angry, disgusted, scared, happy, sad). Faces were displayed until response. There were five practice trials (one for each emotion label) showing cartoon characters from The Simpsons. All children and a subsample of the first 26 of the 57 adults completed the labeling task (*M*_age_ = 19.7 years, SD_age_ = 2.8, age range = 17–27, 16 females). The task was exactly the same for children and adults.

Prior to starting the labeling task, we verified children understood the meaning of the emotion labels. Children were asked for each emotion, “Tell me what happy (*or* sad, etc.) means or when you might feel happy (*or* sad, etc.).” All children provided explanations that were consistent with the meaning of the emotion labels (e.g., for happy: “if something goes your way or if you get something that you like”). Children were then read five brief stories from Widen and Russell (2002) depicting scenarios that would be likely to elicit each of the five emotions, and asked “How do you think [the child in the story] is feeling?” All children verbalized the correct emotion label or a synonym for that emotion for each story (e.g., some children gave the label “afraid” instead of “scared” for the fear story).

### Authenticity Discrimination Task

**FIGURE 2:**
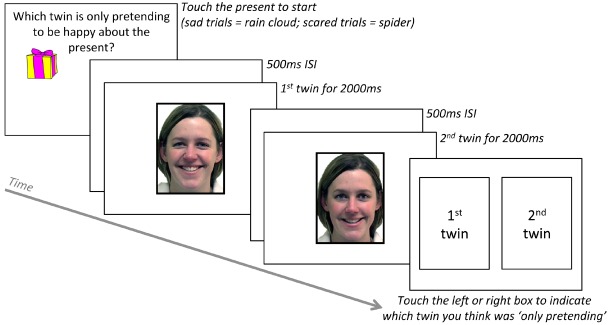
**Trial sequence for authenticity discrimination task (happy example shown here; correct answer = 2nd twin was only pretending). Adapted from McLellan et al. (2010)**.

The authenticity discrimination task is illustrated in Figure 2. Two images of the same person were presented one after the other for 2000 ms each, with a blank interstimulus interval of 500 ms. One image showed a genuine happy, sad, or fear expression and the other image showed a pretend version of the same emotional expression. Participants were instructed that the two images were of twins, and that “The twins are playing a trick on you. One twin really feels happy, scared, or sad, but the other is only pretending! Your job is to decide which twin is only pretending.” The task was exactly the same for children and adults, with the exception that, following these initial instructions, children were also asked what “pretending” means. All children were able to give a synonym (e.g., “faking”) and/or an example of pretend behavior.

Each trial started by asking, “Which twin is only pretending to be happy about the present (*or* sad about the rain, *or* scared of the spider)?” To ensure participants were looking at the screen they were required to press an object to start each trial (happy = present; sad = rain cloud; fear = spider). After viewing the two face images, participants responded by touching one of two boxes shown side-by-side onscreen, labeled “‘1st twin” and “‘2nd twin,” to indicate which twin they thought was only pretending to be happy, sad, or scared. Prior to starting the task, all participants completed three practice trials (one each for happy, sad, and scared) showing cartoon characters from The Simpsons. The “twin” images were shown consecutively, rather than together, to ensure participants had the same amount of time to scan each face. The order of genuine and pretend versions of each emotion was counterbalanced across trials (e.g., so the 1st twin displayed genuine happiness in two of the four happy trials, and the 2nd twin displayed genuine happiness in the other two trials). Trial order was randomized for each participant (trials per emotion = 4, total trials = 12).

### Intensity Rating Task

Intensity ratings were from the same subsample of adults who completed the emotion labeling task (*n* = 26). Participants were instructed, “Your next task is to rate the intensity of each facial expression, from weak to strong.” Each face was presented individually onscreen, in random order, with the statement, “Please rate the intensity of this facial expression,” and a scale numbering from 1 (labeled “weak”) to 9 (labeled “strong”). (Note the intensity rating task also included the anger and disgust expressions).

### Empathy Questionnaires

Empathy was measured for children using the Griffith Empathy Measure (GEM; Dadds et al., 2008) and in adults using the Basic Empathy Scale (BES; Jolliffe and Farrington, 2006). These measures were selected because: (1) they each have a two-factor structure in which one factor taps affective empathy and the other cognitive empathy, and (2) they are well matched across the child and adult measures in terms of the number of items that refer to negatively valenced emotions, positively-valenced emotions, and to “feelings” generally without specifying valence (see Table 1).

**Table 1:** **Number of items by valence for the affective and cognitive subscales of the GEM (children) and the BES (adults)**.

	**Griffith empathy measure (GEM)**		**Basic empathy scale (BES)**	
	**No. of items**	**Example**	**No. of items**	**Example**
**Affective subscale**				
Negative valence	7	My child gets upset when another person is acting upset.	7	After being with a friend who is sad about something, I usually feel sad.
Positive valence	1	My child acts happy when another person is happy.	0	–
Unspecified valence	1	My child seems to react to the moods of people around him/her.	4	I get caught up in other people’s feelings easily.
**Cognitive subscale**				
Negative valence	4	When I get sad my child doesn’t seem to notice.	4	When someone is feeling “down” I can usually understand how they feel.
Positive valence	2	My child doesn’t understand when other people cry out of happiness.	3	I can usually work out when people are cheerful.
Unspecified valence	0	–	2	I can often understand how people are feeling even before they tell me.

The GEM is a 23-item parent report measure, with items scored from –4 (strongly disagree) to +4 (strongly agree). Both factors demonstrate good to acceptable reliability (current sample: affective α = 0.81; cognitive α = 0.50; note the cognitive subscale only has six items, and Cronbach’s α tends to underestimate reliability when there are small numbers of items; Schmitt, 1996). Concerning validity, parent ratings on the GEM have been demonstrated to correlate positively with direct observations of children’s empathic behavior and with questionnaire measures of prosocial behavior (Dadds et al., 2008).

The BES is a 20-item self-report measure that uses a 5-point Likert scale, ranging from strongly disagree to strongly agree. Both factors demonstrate good reliability (current sample: affective α = 0.89; cognitive α = 0.77). Concerning validity, each factor correlates as would be expected with other questionnaire measures of empathy and of personality traits (e.g., positive correlation with agreeableness; Jolliffe and Farrington, 2006; Baldner and McGinley, 2014).

## Results

Given that we had no specific predictions for individual emotions, all significance tests throughout the Results that report individual emotions are *post hoc* and all *p*-values are Bonferroni corrected (for the three emotions). Also, all significance tests are two-tailed.

**FIGURE 3:**
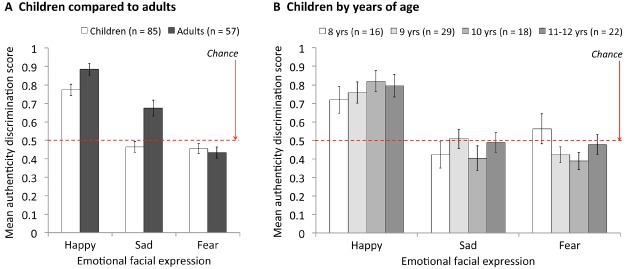
**Mean authenticity discrimination scores for (A) children compared to adults and (B) children by years of age.** Error bars = ± 1 SEM (Standard Error of the Mean).

Authenticity discrimination scores (Figure 3) were calculated as proportion correct (i.e., the proportion of trials on which the participant correctly chose the pretend expression as the one “just pretending”). Initial ANOVA revealed a two-way interaction between age group (adult, child) and face emotion (happy, sad, fear), *F*(2,280) = 5.87, MSE = 0.08, *p* = 0.003, which established the pattern of authenticity discrimination ability across the three emotions was significantly different for adults and children. Thus we analyze adults and children separately.

### Adults

Results are displayed by the dark bars in Figure 3A. A one-way ANOVA on adults’ authenticity discrimination scores revealed a significant effect of face emotion, *F*(2,112) = 42.28, MSE = 0.07, *p* < 0.001, which established that adults’ authenticity discrimination ability differed across the three emotions. Follow-up *t*-tests revealed that adults’ ability to discriminate the authenticity of expressions was significantly better for happy than for sad expressions, *M*_happy_ = 0.89, *M*_sad_ = 0.68, *t*(56) = 4.18, *p* < 0.001, and was also significantly better for sad than for fear expressions, *M*_fear_ = 0.43, *t*(56) = 5.06, *p* < 0.001.

It was also theoretically important to establish whether authenticity discrimination was above chance (0.5). Using one-sample *t*-tests we found that, consistent with previous studies using similar stimuli (e.g.,McLellan et al., 2010, 2012; Johnston et al., 2011; Douglas et al., 2012; McLellan and McKinley, 2013), adults were able to successfully discriminate the authenticity of happy, *t*(56) = 12.08, *p* < 0.001, and sad expressions, *t*(56) = 4.09, *p* < 0.001. For fearful expressions, authenticity discrimination was slightly below chance but not significantly so, *t*(56) = 2.22, *p* = 0.061. Overall, these results indicate that adults could not discriminate the authenticity of the fear expressions, but could discriminate the authenticity of the happy and sad expressions, and that they were better at this for happy than for sad.

### Children

Results are displayed by the light bars in Figure 3A. A one-way ANOVA on children’s authenticity discrimination scores revealed a significant effect of face emotion, *F*(2,168) = 32.37, MSE = 0.09, *p* < 0.001, which established that authenticity discrimination ability differed across the three emotions. Follow-up *t*-tests revealed that, like adults, children showed better ability to discriminate the authenticity of happy than of sad expressions, *M*_happy_ = 0.77, *M*_sad_ = 0.46, *t*(84) = 6.36, *p* < 0.001. Unlike adults, however, there was no significant difference between children’s authenticity discrimination scores for sad and fear expressions, *M*_fear_ = 0.46, *t*(84) = 0.227, *p* = 1.0.

Comparison of children’s authenticity discrimination scores to chance (0.5) for each emotion using one-sample *t*-tests also showed that, like adults, children were able to successfully discriminate the authenticity of happy, *t*(56) = 8.93, *p* < 0.001, and not of fearful expressions, *t*(56) = 1.61, *p* = 0.224, but that, unlike adults, children were unable to discriminate the authenticity of sad expressions, *t*(56) = 1.12, *p* = 0.474.

**FIGURE 4:**
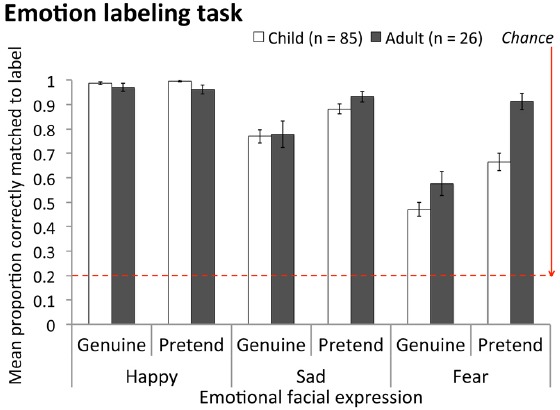
**Mean proportion correct for emotion labeling task for children compared to adults, for genuine and pretend happy, sad and fear expressions.** Labeling items were scored as correct if participants selected the label that McLellan and colleagues had ascribed to a stimulus (personal communication, 2011). Note, overall fear labeling accuracy was lower than for other emotions for both age groups, but this is a typical finding (e.g., Palermo and Coltheart, 2004). Error bars = ± 1 SEM.

Finally, it was also important to compare children to adults. This must be done separately for individual emotions (due to the original emotion × age group 2-way interaction, which indicates developmental improvement varies across emotions). Results showed there was no age-related change in authenticity discrimination accuracy for fear expressions, *t*(140) = 1.56, *p* = 0.602, but that children performed significantly more poorly than adults for happy and sad expressions, happy: *t*(140) = 2.46, *p* = 0.015; sad: *t*[106.1(equal variances not assumed) = 4.04, *p* < 0.001]. Importantly, children’s poorer authenticity discrimination for happy and sad expressions could not be explained by any inability to label these expressions (Figure 4). Children’s labeling accuracy for sad expressions was statistically equivalent to that of adults, *M*_child_ = 0.83, *M*_adult_ = 0.86, *F*(1, 109) = 0.574, MSE = 0.060, *p* = 0.450, and for happy expressions was slightly better than that of adults, *M*_child_ = 0.99, *M*_adult_ = 0.97, *F*(1, 109) = 6.95, MSE = 0.004, *p* = 0.010, irrespective of whether expressions were genuine or pretend (no significant interaction between age group and expression authenticity for happy or sad expressions, both *p*s > 0.222).

Overall, results for children showed they were able to discriminate the authenticity of happy expressions, but not of sad (or fearful) expressions, and that even in the case of happy expressions children did not perform as well as adults.

### Does Authenticity Discrimination Improve from 8 to 12 Years of Age?

Figure 3B illustrates that, for all three emotions, children showed no improvement in their ability to discriminate the authenticity of expressions with age (i.e., the 8-year-old white bars on the left for each emotion are comparable with the darker grey bars for older children on the right). Supporting this conclusion, linear trend analysis on mean authenticity discrimination score across age in years (8 year olds, 9 year olds, 10 year olds, and 11- and 12-year-olds combined for sufficient sample size) showed no significant change with age for any of the three emotions, happy: *F*(1,81) = 0.98, MSE = 0.08, *p* = 0.326; sad: *F*(1,81) = 0.11, MSE = 0.08, *p* = 0.739, fear: *F*(1,81) = 1.25, MSE = 0.06, *p* = 0.266.

### Intensity

Our results show that children are not able to discriminate the authenticity of sad expressions at all, and are less able than adults to discriminate the authenticity of happy expressions. This raises the question of whether children and adults use different strategies to discriminate authenticity. We tested the extent to which children rely on expression intensity to judge authenticity, as opposed to also using additional cues in the face (e.g., reliable AUs; Thibault et al., 2009, 2012), across all three of the expressions we tested (happy, sad, fear; note the previous studies of Thibault et al., 2009, and Del Giudice and Colle, 2007, examined variations in intensity within happy only). Note that our method and analyses are designed to establish (1) the contribution of intensity to authenticity discrimination and (2) *if* additional cues contribute. They are not intended to establish *what* these additional cues are.

**FIGURE 5:**
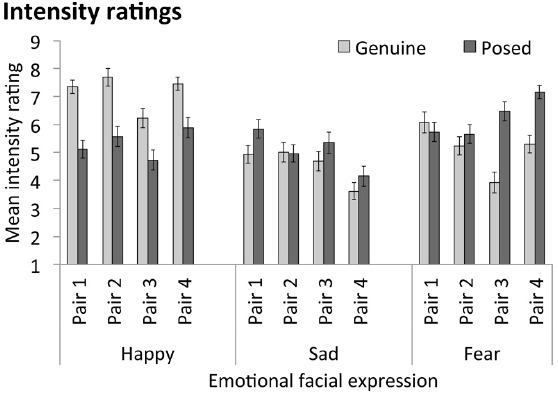
**Mean intensity ratings (*n* = 26) for individual stimulus pairs (e.g., Happy Pair 1 = genuine and posed happy expressions shown by the same person).** Error bars = ± 1 SEM.

We first examined the mean intensity ratings for our stimulus items from the three emotions, shown in Table 2 averaged across emotions and in Figure 5 for individual stimulus pairs. These values are potentially consistent with the idea that children (and adults) judge authenticity based on the intensity of the expression, such that they perceive the more intense expression of each trial pair as the more genuine, and the less intense as less genuine (more likely to be pretend). Specifically, for happy—the expression for which children were able to reliably discriminate authenticity—comparison of mean ratings showed the genuine items we used were on average significantly more intense than the pretend items, *t*(25) = 7.65, *p* < 0.001. For sad, however, the genuine items and pretend items did not differ significantly in average intensity, *t*(25) = 1.54, *p* = 0.137; and for these stimuli children were not able to discriminate authenticity above chance (while adults could). Finally, for fear, the *pretend* items were significantly more intense overall than the genuine items, *t*(25) = 5.55, *p* < 0.001; and for these stimuli even adults could not discriminate authenticity above chance (indeed, they showed a trend in the opposite direction, i.e., toward perceiving the *pretend* item as more authentic than the genuine one).

**Table 2:** **Mean intensity ratings (*n* = 26 adults) for genuine and pretend versions of each emotional expression, with SDs in parentheses**.

**Genuine**			**Pretend**		
**Happy**	**Sad**	**Fear**	**Happy**	**Sad**	**Fear**
7.18	4.55	5.13	5.32	5.07	6.25
(1.2)	(1.2)	(1.0)	(1.6)	(1.2)	(1.0)

Scale = 1 to 9, with higher number indicating the expression is stronger (more intense). Scores are averaged over the four items in each category (e.g., the four genuine happy face items).

**FIGURE 6:**
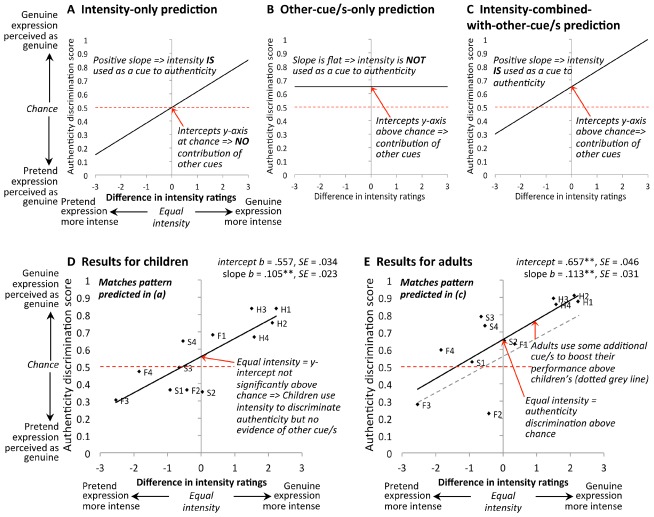
**(A–C)** show predicted pattern of results if, to discriminate expression authenticity, participants **(A)** rely exclusively on intensity, or **(B)** use some other cue/s and *not* intensity information, or **(C)** combine intensity information with other cue/s. **(D)** Results for children (*n* = 85) indicate they use intensity information with no significant contribution of other factors. **(E)** Results for adults (*n* = 57) indicate they also use intensity information, but that they combine intensity with some other cue/s to boost their performance above that of children. “Difference in intensity ratings” is the difference in mean rating, for each stimulus pair (*n* = 12 pairs), averaged across 26 adult participants (e.g., mean intensity rating for a genuine happy stimulus face minus the mean intensity rating for the corresponding pretend happy stimulus with which it appeared on the 2-AFC trial). Authenticity discrimination scores were averaged across participants for each stimulus pair. H1 = happy stimulus pair number 1, H2 = happy stimulus pair number 2, and so on for sad (S) and fear (F).

Taking this intensity analysis one step further, we then examined correlations between authenticity discrimination performance and intensity of individual items. Note our use of paired stimuli (for 2-AFC) for the authenticity trials requires a somewhat complicated way to analyze the data (i.e., we cannot just plot discrimination accuracy against intensity of *the* face, since there were two faces presented on each trial). In Figure 6, we illustrate the format of our data plots. On the x-axis, we plot the *difference* in mean intensity ratings for each genuine-pretend pair. On this scale: a score of zero indicates that the genuine and pretend items on the trial were of equal intensity; a score to the right of zero indicates that the genuine face was more intense than the pretend face; and a score to the left of zero indicates that the pretend face was more intense than the genuine face. On the y-axis, we plot authenticity discrimination accuracy (mean across participants), for each individual face pair (12 pairs in total).

In Figures 6A–C, we illustrate how various results outcomes would correspond to evidence of using different types of strategies. In particular, we test whether a given age group is using: (a) intensity *only* (Figure 6A); (b) other strategies *only* (Figure 6B), such as making use of the reliable AUs (AU6 for happy and AU1+4 for sad) that are present in the genuine versions and absent in the pretend versions, or making use of affective empathy responses; or (c) a *combination* of intensity and other strategies (Figure 6C).

First, in Figure 6A, if intensity is an important driver of which member of the pair is perceived as authentic, we would expect to observe a positive-slope relationship. Here, pairs in which the genuine face is the more intense item would tend to produce higher authenticity decision accuracy (i.e., participants are more likely to correctly perceive the genuine face as the genuine one), and pairs in which the pretend face is the more intense item tend to produce lower authenticity decision accuracy (i.e., participants are more likely to incorrectly perceive the pretend face as the genuine one). Moreover, if participants are using *only* intensity to determine authenticity, then we would expect the line of best fit to pass through (0, 0.5): that is, when the two faces in the stimulus pair are equal in intensity (zero on the x-axis), we would expect participants to perform at chance (0.5) in authenticity discrimination.

Second, in Figure 6B, if only other strategies drive percepts of authenticity, with no role for intensity, then we would observe a flat line relationship with the line of best fit set at above-chance discrimination (y-intercept significantly above 0.5 on the authenticity discrimination scale). This is because the lack of a role for intensity would leave no association with that variable (i.e., a flat slope), and the contribution of the other factor/s would give above-chance discrimination (including when intensity is equal for the genuine and pretend faces, i.e., at the y-axis where intensity difference is zero).

Finally, if both intensity *and* other strategies were being used in combination, we would expect the pattern illustrated in Figure 6C. Here, there is a positive-slope relationship indicating a contribution of intensity, and simultaneously an above-0.5 y-intercept value indicating a contribution from other factor/s. Note this is different from the pattern in Figure 6A, in which the y-intercept is 0.5 indicating authenticity discrimination is related *exclusively* to intensity.

We present the actual results in Figure 6D (children) and Figure 6E (adults). For both age groups, authenticity discrimination was significantly positively related to expression intensity (slope of regression line for children: *b* = 0.105, β = 0.823, *t*(11) = 4.58, *p* = 0.001; for adults: *b* = 0.113, β = 0.756, *t*(11) = 3.65, *p* = 0.004), arguing that both children and adults used intensity as a cue to authenticity across the three expressions. Moreover, comparison of slopes across the two age groups indicated that there was no evidence of any difference in children’s compared to adults’ sensitivity to intensity; that is, the child slope of *b* = 0.105 was not significantly less steep than the adult slope of *b* = 0.113, *t*(20) = 0.20, *p* = 0.838 (parallel slopes shown in Figure 6E).

Where children differed from adults was in the y-intercept. For children, when the genuine and pretend expressions in the pair were of equal intensity, the regression line intercepted the y-axis at a point not significantly different from chance levels of authenticity discrimination [*intercept* = 0.557, *t*(11) = 1.68, with *p* = 0.122 for comparison to chance value of 0.5]. Thus in the absence of diagnostic intensity information children were unable to discriminate authenticity above chance (consistent with the earlier analysis for sad-expression items treated as a group). In comparison, Figure 6E shows that the regression line for adults is moved up relative to children’s, and crosses the y-axis significantly above chance levels of authenticity discrimination [*intercept* = 0.657, *t*(11) = 3.41, *p* = 0.006 for comparison to chance value of 0.5]. This result argues that adults used, in addition to intensity, some other cue or cues to improve their discrimination of authenticity.

While the size of our stimuli set for the intensity ratings was small (*n* = 12 stimulus pairs) and we only used adult’s estimates of intensity differences, overall these analyses are consistent with the view that children’s percepts of authenticity were driven primarily by intensity of the expression, while adults judge authenticity using intensity in combination with other factors. Concerning the nature of these other factor/s, note that our adult results do not directly demonstrate that adults used the presence versus absence of reliable AUs, although the results are at least consistent with this view in that our stimuli had reliable AUs present in the genuine version (and absent in the pretend version) for the two emotions that adults could discriminate above chance (happy and sad), but not necessarily for the emotion adults could not discriminate (fear). For children, however, our results do directly support the view that this age group did *not* use the reliable-AU combination of AU1+4 as a cue to authenticity for sad faces: if they did so, then their mean performance for sad faces would have to be above chance, which was not the case (see Children).

### Empathy

We next examined correlations between individual differences in empathy and authenticity discrimination ability, for those emotions where performance was above chance. Reliability analyses are reported in Table 3 and correlational results in Table 4. Note that for conditions where mean authenticity discrimination was at chance (fear for adults, sad and fear for children), individual differences in performance are not meaningful (i.e., they are taken to reflect merely guessing), and so correlations with empathy are not reported.

**Table 3:** **Reliability for measures used in individual differences analyses**.

	**Number of items**	**Reliability coefficient^1^**
**Children (*n* = 85)**		
Empathy		
GEM_affective_	9	0.81
GEM_cognitive_	6	0.50
GEM_negative–valence_	11	0.84
GEM_positive–valence_	3	0.47
Authenticity discrimination		
Happy	4	0.61
**Adults (*n* = 57)**		
Empathy		
BES_affective_	11	0.89
BES_cognitive_	9	0.77
BES_negative–valence_	11	0.81
BES_positive–valence_	3	0.75
Authenticity discrimination		
Happy	4	0.75
Sad	4	0.66

^1^For scales with >3 items we report Cronbach’s α; however, α tends to underestimate reliability when there are small numbers of items (Schmitt, 1996) so for the 3-item scales we report the Spearman-Brown coefficient (Eisinga et al., 2013).

**Table 4:** **Correlations between empathy and authenticity discrimination scores (for emotions where mean performance was above chance)**.

	**All participants^1^**			**Females only**		
	**Children (*n* = 85)**	**Adults (*n* = 57)**		**Children (*n* = 46)**	**Adults (*n* = 40)**	
	**Happy^1^**	**Happy^1^**	**Sad**	**Happy^1^**	**Happy^1^**	**Sad**
Affective empathy	0.053	0.309**	–0.119	0.084	0.277*	–0.255
Cognitive empathy^2^	–0.004	0.352**	–0.108	0.015	0.334**	–0.118
Positively-valenced^3^	0.038	0.383**	–0.067	–0.018	0.346*	–0.011
Negatively-valenced^2,3^	0.083	0.342**	–0.106	0.105	0.352*	–0.155

*Empathy was measured in children using the GEM and in adults using the BES. Correlations for fear for both age groups and for sad for children are not presented because mean performance was at chance and, correspondingly, internal reliability was extremely low. ^1^Kendall’s ** is reported for correlations involving happy authenticity discrimination scores, because this variable was strongly negatively skewed. Pearson’s *r* is reported for all other correlations. ^2^Correlations with the GEM cognitive subscale and the GEM positively-valenced subscale should be interpreted with caution due to the low reliability of these subscales, but are presented here for completeness. ^3^Positively-valenced and negatively-valenced scores were calculated by summing together all items from the *total* scales (because there were too few items if we did so for the affective and cognitive subscales separately) that referred to positive and negative emotions respectively (see* **Table 1**), excluding all items for which emotional valence was not specified. *p < 0.05, **p < 0.01.

For adults, there were significant positive correlations (i.e., higher empathy was associated with better ability to discriminate authenticity) for happy expressions. This was true for both affective empathy, τ = 0.309, *p* = 0.004, and cognitive empathy, τ = 0.352, *p* = 0.001 (note all correlations involving happy in this article report the non-parametric Kendall’s τ due to a skewed distribution of authenticity discrimination scores for this expression). In an additional analysis, we divided the adult empathy questionnaire (BES) into items that referred to negative emotions and positive emotions (excluding items in which the emotional valence was not specified; see Table 4). This was because Manera et al. (2013) reported that *better* authenticity discrimination was related to susceptibility to emotional contagion (one aspect of empathy; Maibom, 2012) only for contagion from *negative* emotions, while susceptibility to emotional contagion from *positive* emotions was related to *worse* authenticity discrimination (note the results were for happy-face authenticity only; other expressions were not tested). However, in the present study we found that both negative-valence and positive-valence BES scores showed significant positive correlations with authenticity discrimination for happy expressions (BES_negative–valence_: τ = 0.342, *p* = 0.002; BES_positive–valence_: τ = 0.383, *p* = 0.001). That is, we found in adults that better authenticity discrimination of happiness was associated with greater BES empathy, *irrespective* of the emotional valence of measurement items.

In contrast, for children we found no significant correlations with empathy (Table 4), specifically including trivially small correlations for happy (i.e., the expression for which significant correlations were present for adults). Note this lack of correlation cannot be attributed to uninteresting explanations such as lack of range: the children’s happy-face authenticity scores had if anything more range than the adults’ (SD_children_ = 0.28, SD_adults_ = 0.24), and the children’s empathy scores covered a wide range of values compared to norms (for total GEM, *M* = 34.32, SD = 18.94, compared to *M* = 35.03, SD = 21.7 for *n* = 1034 7–10 year olds; Dadds et al., 2008).

**FIGURE 7:**
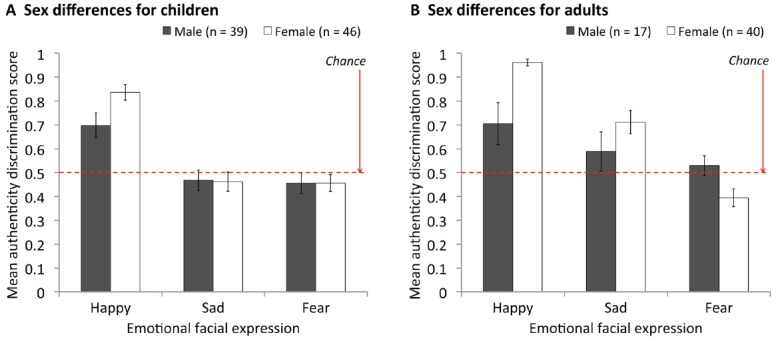
**Mean authenticity discrimination scores comparing males and females for (A) children and (B) adults.** Error bars = ± 1 SEM.

Finally, we examined whether sex differences might play a role in the empathy correlations found in adults. This issue arose because (a) empathy was positively related to being female in our adult sample (affective empathy: *r* = 0.587, *p* < 0.001; cognitive empathy *r* = 0.299, *p* < 0.05; replicating previous findings, for review see Eisenberg and Lennon, 1983), and (b) at the same time, we found that authenticity discrimination was also better in females. Including participant sex in a global ANOVA [sex × facial emotion × age group (children vs. adults)] on authenticity scores revealed a significant interaction between sex and face emotion, *F*(2, 276) = 7.04, MSE = 0.076, *p* = 0.001. This interaction is illustrated in Figure 7, where it can be seen that females showed an advantage over males in authenticity discrimination for happy faces, but not sad or fear faces. Collapsing over age group [noting that the ANOVA showed no significant interactions involving sex and age group: 2-way sex × age, *F*(1, 138) = 0.56, MSE = 0.056, *p* = 0.456; 3-way sex × age × emotion, *F*(2, 276) = 2.29, MSE = 0.076, *p* = 0.103], there were no sex differences for either sad or fear expressions, both *p*s > 0.498. However, for happy, the female advantage was significant, *M*_females_ = 0.90, *M*_males_ = 0.70, *t*[75.6(equal variances not assumed) = 3.94, *p* < 0.001]. This raises the possibility that the significant empathy correlations for adults were in fact driven by sex differences. To rule out this possibility, we re-ran correlations using only female participants. (There were insufficient males in our adult sample to look at males separately). These female-only analyses (right side of Table 4) replicated the finding of a significant relationship between authenticity discrimination for happy expressions and empathy in adults (affective empathy, τ = 0.277, *p* = 0.040, cognitive empathy, τ = 0.334, *p* = 0.015), and not in children, indicating that our empathy findings were not due to sex effects.

Overall, empathy results indicate that individual differences in empathy were correlated with ability to discriminate authenticity for happy expressions in adults, but not in children, and not for sad expressions in adults (with fear correlations in both age groups, and sad correlations in children, not analysable due to the chance performance).

## Discussion

The present study is the first to test children’s ability to discriminate the authenticity of facial expressions for any basic emotions beyond happy, using stimuli in which the genuinely-felt or posed nature of the underlying emotion is known from the self-reports of the person appearing in the photograph. Overall, our results indicate that 8–12-year-olds have some ability to discriminate authenticity in facial expressions, but are immature relative to adults both in their performance level and in the strategies they use to achieve that performance. For happiness, in which genuinely-felt expressions were more intense than posed expressions, children were able to successfully discriminate expression authenticity from the youngest age tested (i.e., 8 year olds), but they did not perform as well as adults, and showed no improvement in this ability over the 8–12 year old age range. When genuinely-felt facial expressions were not more intense than the posed versions for our sad and fear stimuli, then children failed to discriminate authenticity, whereas adults could for sad expressions, arguing that the sad expressions included some other cue or cues to authenticity that children failed to use. Overall, children appeared to judge authenticity exclusively based on intensity of the expression, for all three expressions. In contrast, adults used intensity combined with other factor/s, which for happy expressions may include empathic responses.

### Ruling Out Uninteresting Interpretations of Children’s Poor Performance

Before proceeding to discuss theoretically interesting interpretations of the differences between children and adults, it is important to rule out uninteresting possibilities. This arises particularly for the differences between children and adults in overall performance level (rather than for evidence concerning different strategies).

First, children’s poorer authenticity discrimination, found for happy and sad, could not be explained by any inability to label the expressions. Children were as accurate at labeling happy and sad expressions as adults.

Second, children’s poor performance cannot be explained by failure to understand the task instructions. We verified that all children understood the meaning of pretending as distinct from genuinely-felt emotion, plus children’s above-chance performance for happy expressions shows they were able to correctly follow the instructions to choose the face that was “just pretending.”

Finally, it is important to evaluate whether children’s poor performance might be attributed to factors associated with general cognitive development that might lower laboratory task performance independent of difficulties in perceiving authenticity. As argued previously for other face tasks (e.g., McKone et al., 2012), such factors could include greater distractibility in younger children (i.e., poorer concentration on the task) or, in the present design, difficulties with remembering the *order* in which two items were presented. For the present findings, a number of observations argue against such an interpretation. Concerning order, previous studies have shown that the type of sequential task we used (participants have to remember which item was first and which second, and indicate their choice after a short delay) can easily be performed by children even at the younger end of our age range, when the perceptual discrimination between the two items is straightforward (e.g., accuracy >90% for 9-years-olds in remembering the order of faces displaying happy, sad and fear expressions; Pollak and Kistler, 2002). Concerning distractibility and other attention-related factors, if these factors were responsible for our children’s poor performance, then we would have expected to see authenticity-task performance improve significantly across the 8–12 year age range (because it is plausible that distractibility decreases across this age range); yet this was not the case. And, further, distractibility and other attention-related factors would be expected to lower children’s slope in our plots of authenticity performance against relative intensity (Figure 6D)—because lapses in attention would increase random errors in responses, having the result of pushing both ends of the line toward chance (0.5) thus decreasing its slope—yet, again, this was not the case (i.e., children’s sensitivity to intensity was no weaker than adults’).

Overall, we argue our that our results do not reflect difficulties with task demands, but instead indicate that children aged 8–12 years have poor actual ability to discriminate authenticity in the faces.

### What Cues Do Children Use to Discriminate Authenticity?

Our results argue that 8–12 year old children use immature strategies relative to adults to determine authenticity of facial emotion, relying only on intensity and not other additional cues.

Concerning intensity, we found that the children were equally as sensitive to intensity as a cue to authenticity as adults. This early sensitivity to intensity is in agreement with the two previous studies (Del Giudice and Colle, 2007; Thibault et al., 2009), which showed that children use intensity to judge facial expression authenticity—and rely on it as much or more so than adults—from as young as 4 years of age. Importantly, these previous studies tested only happy faces, and the present study has replicated and extended this result to also include negative-valence facial expressions.

Beyond intensity, we found no evidence that 8–12-year-olds could use any additional strategies. Concerning reliable-AUs, for sad AU1+4 was present in the genuine versions of our stimuli and absent in the pretend versions, yet children could not discriminate authenticity for sad faces above chance (contrasting with evidence that adults can use AU1+4 to determine authenticity; McLellan et al., 2010; but see Mehu et al., 2012). With respect to happy, our correlations with intensity across all three expressions (Figure 6D) are not inconsistent with ideas that children may potentially use AU6 but (in contrast to the conclusion of Thibault et al., 2009) suggest AU6 affects children’s authenticity judgements only to the extent that *the presence of AU6 increases the intensity* of happy expressions. The idea that 8–12-year-olds do not yet use reliable-AUs effectively is also consistent with results for happy from Del Giudice and Colle (2007), who found 8-year-olds, unlike adults, interpreted a facial action that is similar to AU6, AU7 (the “lid tightener”), as signaling authentic happiness. We also found no suggestion that children used any strategies related to empathy (as potentially used by adults).

Overall, the results of the present study converge with previous findings to support a theoretical view in which intensity is the primary or only cue that elementary or primary-school aged children use to judge facial expression authenticity, and that the difference between adults and children in authenticity-discrimination ability arises because adults develop extra strategies in addition to intensity that emerge later in development (i.e., after 12 years of age).

An important question, then, becomes *why* is it that intensity would emerge earlier during development than other strategies? Concerning why intensity is learned *early*, one possibility might be that (a) intensity might have particular real-world value as an authenticity cue for happy expressions (i.e., more intense smiles are more likely to be genuine; Hess et al., 1995; Krumhuber and Manstead, 2009), combined with (b) children might have more opportunity to learn about the authenticity of happy than other emotions in early life, due to explicit instruction from parents (even young children are taught to pretend happiness in keeping with social norms, e.g., “smile for the camera”) and/or having regular opportunities to observe genuine-pretend contingencies (i.e., their parents display intense happy expressions in response to something funny, and less intense happy expressions when politely greeting a disliked relative). Indeed, it may then be that initial learning of the value of intensity for determining genuineness of happy faces is extended by children (and adults) to a use of this cue for other emotions including, potentially, to emotions where intensity is in fact *not* a valid cue to authenticity (as for our sad and fear stimuli in the present laboratory study; and as indeed could occur in the real world where, for example, we know of no evidence as to whether genuinely-felt sad or fear expressions are typically more, or less, intense than their pretend counterparts).

Concerning why it is that other facial cues to authenticity (e.g., reliable-AUs, arousal cues) are learned *later* in development, a plausible possibility is that these are simply less physically obvious than intensity, resulting in young children either failing to *perceive* these more subtle cues or, perhaps, correctly perceiving them but failing to have learnt what they *mean* (e.g., 6–7 year olds do not consciously know the AU6 display rule; that is, “wrinkles around the eyes mean someone is really feeling happy,” Gosselin et al., 2002b).

### What Additional Cues Do Adults Use to Discriminate Authenticity?

Our results argue that adults use extra strategies in addition to intensity information. Concerning the nature of these strategies, the present study investigated correlations with empathy, and found evidence potentially consistent with adults using empathy-related strategies for determining authenticity of happy faces (but not sad faces). Concerning the correlation with affective empathy, theoretically, we suggest that participants might be able to use awareness of their own empathic response to the faces (e.g., Manera et al., 2013) to help judge authenticity. This is on the assumption that affective responses to genuine expressions might be stronger than to pretend ones, and this increased response to genuine faces might be greater in individuals with high affective empathy than in individuals with lower affective empathy. This reasoning predicts a positive correlation between affective empathy and authenticity discrimination ability, as we found. Concerning the correlation with cognitive empathy, then a positive correlation—as we found—is predicted if we assume that cognitive empathy might include knowledge of what physical cues are indicative of expression authenticity (e.g., that AU6 signals genuine happiness). (Importantly, we note that both these ideas make an assumption about the direction of causation, namely that empathy is causing strategies that assist with authenticity discrimination. It is, of course, equally possible that the correlation between the two variables could reflect an opposite-direction causality, in which individuals who are better at recognizing authentic emotions in others’ faces go on to develop higher empathy as a result, at least by the time they are adults).

Regarding other possible strategies, our results suggest that adults use reliable-AU1+4 for sad, given that: this AU was present in our genuine stimuli and absent from the posed versions; and adult authenticity performance was above chance at the same time that intensity of the genuine and pretend versions was equal and there was no association with empathy. This adds to earlier evidence that adults use reliable-AUs (AU6 for happy, Del Giudice and Colle, 2007, Thibault et al., 2012) or proposed reliable-AUs for other emotions (e.g., AU15 for panic fear, Mehu et al., 2012) to judge authenticity. Additionally, adults may also use other physical cues within faces that have been proposed to differ between genuine and posed expressions (e.g., signs of physical arousal such as pupil dilation or skin “blushing,” Levenson, 2014; or symmetry, Ekman, 2003).

### Why Can’t Even Adults Discriminate the Authenticity of the Fear Expressions?

In our study, neither adults nor children could discriminate the authenticity of the fearful expression stimuli. This finding agrees with one of the two previous (adult) studies of fear stimuli generated using the same Miles/McLellan method (Douglas et al., 2012, obtained A’ = 0.48 where chance is 0.5) and is not very different from the other (which found significant but weak discrimination ability; A’ = 0.61, McLellan et al., 2010). We suggest two possible explanations for poor authenticity discrimination of the fear expressions.

One idea is that, while it may be adaptive to discriminate authenticity of most emotions (including in the present context, happy and sad), for fear “the negative consequences of failing to detect (fear) and then avoid (the cause of that fear) perhaps render even close approximations of fear signals as real” (McLellan et al., 2010, p. 1285). That is, it may be that it is adaptive to treat all fear expressions as if they are genuine (i.e., in everyday life, this could allow a person to rapidly avoid danger without waiting for a more time-consuming analysis of authenticity to be completed).

Alternatively, the inability to tell apart genuine and posed fear could reflect physical characteristics of the particular Miles/McLellan stimuli. These might fail to match real-world genuine fear faces in at least two ways. First, as noted, reliable-AUs for fear have not been empirically validated (although suggestions have been made by Ekman, 2003), and thus it is not known whether the fear stimuli included reliable-AUs (should they exist). Second, the fear faces are probably only modest in terms of the underlying strength of the emotion felt by the person shown in the stimulus photograph, meaning that the potential for the presence of other physical cues to genuineness (particularly arousal cues, i.e., pupil dilation, skin tone changes, etc.) may be limited. This is an intrinsic limitation of any fear face stimuli created in a laboratory setting. It is difficult to invoke a very strong feeling of fear in the lab: for somebody to feel strong fear, they must believe there is real danger, and it is not ethical or practical to, for example, release a tiger into the lab, or to set off a bomb. By comparison, it is much easier to induce strong underlying emotions of happiness in the lab (e.g., there are no ethical problems with making somebody laugh hilariously).

### Limitations

The present study has a number of limitations on scope, with corresponding implications for generalisability of the results. Perhaps most significantly, we used a small sample of stimuli (from McLellan and colleagues, personal communication, 2011). We chose to do this because we are unaware of any other stimulus sets meeting the core criteria we wanted our stimuli to meet: a set containing happy, sad and fear; in which the same model displays both genuine and pretend versions; for which it has been verified by self-report of the people photographed that their underlying emotions were indeed genuinely-felt, or pretended, respectively; and for which FACS coding confirmed the presence of empirically-supported reliable-AU markers in the genuine version (i.e., AU6 for happy and AU1+4 for sad). This small set of stimuli, however, is limited in four important ways. Regarding *intensity*, the genuine sad and fear faces are only moderate in expression intensity (Figure 5), and likely correspond to rather substantially lower intensity of the underlying emotion than would occur in some real world situations (e.g., bereavement; a terrorist attack); thus, we cannot rule out that above-chance authenticity discrimination might emerge for sad and fear expressions in children (and for fear in adults), if the genuine expression reflects a more intense underlying emotion (even if the pretend expression is also high-intensity to match). Concerning *age of the faces*, all images showed adults, and children may have more experience with children displaying genuine versus pretend sadness (e.g., siblings faking tears to get sympathy from a parent), and we also used *static photographs* while real-world facial expressions are dynamic (over a few hundred milliseconds, the expression begins to appear, reaches its maximum intensity, and then disappears again) that include additional physical differences between genuinely-felt and pretend emotions (e.g., genuine expressions have smoother onset and offset than posed ones; e.g., Schmidt et al., 2006). Thus we cannot rule out the possibility that authenticity discrimination could improve if movie-images, or children’s faces, were used. Concerning the *number* of stimuli, it is possible that using a larger set may increase statistical power; while power alone seems unlikely to account for our finding that children could not discriminate sad authenticity above chance (given that the mean performance was 46% with a large number of child participants, i.e., *n* = 85), in the case of the y-intercept from our intensity analysis, this was numerically above 0.5 for children (i.e., y-intercept = 0.56). Potentially, additional stimuli might reduce the SE of the y-intercept value and thus increase the chances of finding evidence that children make some use of strategies additional to intensity (i.e., y-intercept significantly above 0.5).

It is also worth noting that the present study has only tested children’s ability to make *explicit* decisions about authenticity. It would also be of interest to know whether children show differential *implicit* responses to genuine and posed facial expressions (as has been found in adults; e.g., Peace et al., 2006; Miles and Johnston, 2007). Potentially, differences in implicit behavior might emerge earlier than explicit knowledge; for example, even young children might show greater willingness to help a person displaying genuine than posed sadness.

### Conclusion

Our study has provided the first test of authenticity discrimination in children for facial expressions of basic emotions beyond happy (i.e., also sad and fear), including the first examination of use of intensity as a cue to authenticity across this broader range of emotions, plus the first test of relationships with empathy. Our results imply that authenticity discrimination from facial expressions matures surprisingly late in development, specifically some time during the teenage years, with children aged 8–12 having developed adult-like use of expression intensity as a cue to authenticity, but failing to show significant use of skills related to reliable-AUs (for sad) and empathy (for happy). This late maturity of authenticity discrimination ability for facial expressions suggests it will be important in future research to ascertain how its development impacts on social skills, such as friendship formation and maintenance, during the late primary school and teenage years.

### Conflict of Interest Statement

The authors declare that the research was conducted in the absence of any commercial or financial relationships that could be construed as a potential conflict of interest.
